# Correlation of systolic and diastolic blood pressure with echocardiographic phenotypes of cardiac structure and function from three German population-based studies

**DOI:** 10.1038/s41598-023-41571-x

**Published:** 2023-09-04

**Authors:** Julius Nikorowitsch, Ramona Bei der Kellen, Alena Haack, Christina Magnussen, Jürgen Prochaska, Philipp S. Wild, Marcus Dörr, Raphael Twerenbold, Renate B. Schnabel, Paulus Kirchhof, Stefan Blankenberg, Marcello Ricardo Paulista Markus, Jan-Per Wenzel

**Affiliations:** 1 Department of Cardiology, University Heart & Vascular Center Hamburg, University Medical Center Hamburg-Eppendorf, Martinistr. 52, 20246 Hamburg, Germany; 2 German Centre for Cardiovascular Research (DZHK), Partner Site Hamburg/Kiel/Luebeck, Hamburg, Germany; 3 Epidemiological Study Center, Hamburg, Germany; 4 Preventive Cardiology and Preventive Medicine, Department of Cardiology, University Medical Center of the Johannes Gutenberg University Mainz, Langenbeckstr. 1, 55131 Mainz, Germany; 5 Center for Thrombosis and Hemostasis, University Medical Center of the Johannes Gutenberg University Mainz, Langenbeckstr. 1, 55131 Mainz, Germany; 6 German Centre for Cardiovascular Research (DZHK), Partner Site Rhine Main, Mainz, Germany; 7 German Centre for Cardiovascular Research (DZHK), Partner Site Greifswald, Greifswald, Germany; 8 Department of Internal Medicine B, University Medicine Greifswald, Greifswald, Germany; 9 Institute of Cardiovascular Sciences, University of Birmingham, Birmingham, UK; 10 Center for Population Health Innovation (POINT Institute), University Heart and Vascular Center Hamburg, University Medical Center Hamburg-Eppendorf, Hamburg, Germany; 11 Cardio-CARE, Medizincampus Davos, Davos, Switzerland; 12 German Center for Diabetes Research (DZD), Partner Site Greifswald, Greifswald, Germany

**Keywords:** Cardiology, Cardiovascular diseases, Heart failure

## Abstract

Arterial hypertension is considered a risk factor for the development of heart failure. Here we investigate cross-sectional associations of systolic and diastolic blood pressure with subtle functional and morphological changes of left ventricular echocardiographic parameters representing early dysfunction in three representative German population-based studies. We assessed 26,719 individuals without symptomatic heart failure from the Hamburg City Health Study (HCHS, n = 7396, derivation cohort), the Gutenberg Health Study (GHS, 14,715, validation cohort) and the Study of Health in Pomerania (SHIP, 4608, validation cohort). Multivariable linear regression analyses with systolic and diastolic blood pressure as continuous exposure variables were adjusted for common cardiovascular risk factors and antihypertensive medication. Both systolic and diastolic blood pressure were consistently associated with measures of left ventricular hypertrophy (β per standard deviation (SD) for LV mass (g) and systolic blood pressure: 5.09 (p < 0.001); diastolic blood pressure: 2.29 (p < 0.001) in HCHS). Systolic blood pressure correlated with declining diastolic function (β per SD for E/e′: 0.29, p < 0.001 in HCHS) and diastolic blood pressure with declining systolic function (β per SD for LVEF, in %: − 0.15; p = 0.041 in HCHS) in all cohorts. Pending further validation, our results from three independent German population samples suggest differential effects of systolic versus diastolic blood pressure on left ventricular structure and function.

## Introduction

Worldwide, more than 1.2 billion people aged 30–79 years suffer from arterial hypertension^1^. Arterial hypertension is the foremost risk factor for developing heart failure^2^. Newly diagnosed heart failure of any subtype according to Framingham clinical criteria was preceded by arterial hypertension in 91% of subjects in the Framingham Heart Study^3^.

Chronic elevation of blood pressure results in reverse myocardial remodelling including inflammatory and fibrotic processes with consecutive dysfunction of the macro- and microvasculature^4, 5^. Pathophysiologically, hypertensive heart disease begins with left ventricular hypertrophy and diastolic dysfunction^6, 7^. These conditions are considered a result of increased afterload for the left ventricle induced by systolic and diastolic hypertension. Untreated arterial hypertension leads to both cardiac diastolic and systolic dysfunction, finally evinced by manifest heart failure^8^. In patients with heart failure with preserved ejection fraction, only systolic blood pressure was associated with left ventricular hypertrophy while diastolic blood pressure correlated with diastolic dysfunction^9^. Differential interplays of systolic and diastolic blood pressure with cardiac function and morphology even occur during normal aging^10^. Pathological cardiac remodelling including ventricular hypertrophy and diastolic dysfunction often precede symptomatic heart failure^3, 11^. To better characterise the differential role of systolic blood pressure and diastolic blood pressure for the development of heart failure, we investigated associations of systolic blood pressure and diastolic blood pressure with functional and morphological left ventricular echocardiographic parameters in subjects without apparent heart failure from the Hamburg City Health Study (HCHS) validated in the Study of Health in Pomerania (SHIP) and Gutenberg Health Study (GHS).

## Methods

### Study setting—The Hamburg City Health Study (HCHS)

**Figure 1:**
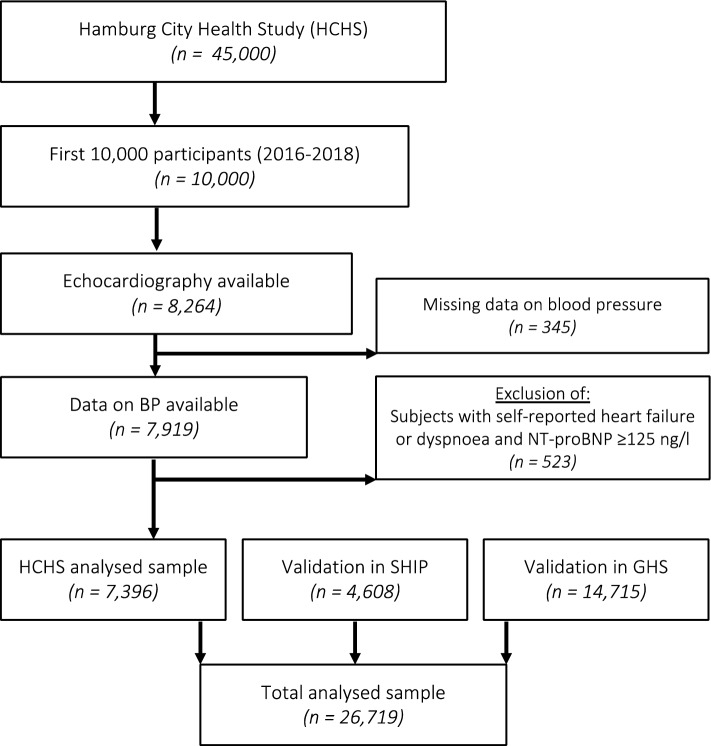
Study PRISMA. From a total of 10,000 subjects, 8264 had available echocardiographic data, 345 were excluded due to missing data on blood pressure. Further 523 subjects were excluded due to self-reported heart failure or dyspnoea (NYHA ≥ II), and NT-proBNP ≥ 125 ng/l. Consequently, 7396 HCHS subjects were included in the study analysis. 4608 subjects from the Study of Health in Pomerania )SHIP) and 14,715 subjects from the Gutenberg Health Study (GHS) were included for validation. In total 26,719 subjects were analysed. *Bp* blood pressure, *HCHS* Hamburg city health study, *NT-proBNP* N-terminal pro b-type natriuretic peptide.

Data from the initial 10,000 participants of the Hamburg City Health Study (HCHS) were examined. The HCHS is a long-term, prospective, population-based cohort study involving a random selection of participants from Hamburg, Germany^12^. Measurements were conducted during a single-day visit at the HCHS Epidemiological Study Centre of the University Medical Center Hamburg-Eppendorf between 2016 and 2018. Out of the first 10,000 participants, 8264 underwent a transthoracic echocardiogram. From this group, individuals with self-reported heart failure or dyspnoea (corresponding to NYHA class ≥ II) in conjunction with N-terminal pro-brain natriuretic peptide (NT-proBNP) levels ≥ 125 ng/l (n = 523), left ventricular ejection fraction (LVEF) ≤ 40% (n = 45), or missing blood pressure data (n = 347) were excluded. A total of 7396 participants were included for subsequent analysis. For validation purposes, data from 4608 individuals from the Study of Health in Pomerania (SHIP) and 14,715 participants of the Gutenberg Health Study (GHS) were assessed (Fig. 1). The detailed study protocols for all three cohorts have been previously published^12–14^.

### Demographics and clinical parameters

Demographic and clinical characteristics were evaluated using standardized interviews carried out by specially trained medical personnel and questionnaires adhering to established standard operating procedures^12^. Blood samples were collected under fasting conditions on the day of examination. NT-proBNP levels were assessed in all patients using stored serum samples (immunoassay by Alere NT-proBNP for ARCHITECT, Abbott Diagnostics, with a measurement range of 8.2–35,000 ng/l). Blood pressure was measured twice using an Omron 705IT device (Omron Electronics GmbH, Mannheim, Germany) on the right upper arm while the participant was seated, following a 5-min rest period; the results were then averaged. Medications classified as antihypertensive drugs encompassed ACE inhibitors, angiotensin II receptor blockers, beta blockers, calcium channel blockers, renin inhibitors, thiazide diuretics and loop diuretics^15^. Atrial fibrillation was considered present if indicated in the questionnaire, diagnosed via a 12-lead electrocardiogram, or both. Diabetes mellitus was determined based on a fasting glucose level of ≥ 126 mg/dl or the use of antidiabetic medications. Coronary artery disease was self-reported and defined as having a history of one or more of the following conditions: myocardial infarction, percutaneous coronary intervention (PCI), or coronary artery bypass surgery.

The local ethics committee of the Medical Association Hamburg (Landesärztekammer Hamburg; PV5131) approved the HCHS. All participants provided written informed consent. The HCHS study's review board approved the study protocol, which conforms to the principles outlined in the Declaration of Helsinki.

### Echocardiographic data

TTE was conducted and analysed by cardiologists and sonographers (technicians) during the baseline visit, following a standardized protocol in accordance with the guidelines set forth by the American Society of Echocardiography (ASE) and the European Society of Cardiovascular Imaging (EACVI)^16^. For continuous quality assessment, every 100th TTE exam was additionally analysed by a second investigator. Qualitative and quantitative image analyses were performed using an off-line workplace (Siemens syngo SC2000 version 4.0 software).

Left-sided volumes and ejection fraction (LVEF) were calculated using the two-dimensional biplane method of disks summation. Left-sided diameters were measured in the parasternal long-axis view. Left ventricular mass was calculated using the Devereux cube formula, based on interventricular septum thickness, LV end-diastolic diameter, and posterior wall thickness. Mitral inflow patterns were assessed in the apical four-chamber view by positioning the pulsed-wave (PW) Doppler sample volume between the mitral leaflet tips. PW tissue Doppler imaging (TDI) e′ velocity was measured in the apical four-chamber view by placing the sample volume at the lateral and septal basal regions. E/E′ was determined as the mean of E/E′ septal and E/E′ lateral.

### Validation cohorts: The Study of Health in Pomerania (SHIP) and Gutenberg Health Study (GHS)

SHIP is a population-based investigation conducted in Pomerania, Germany. The study's design and recruitment strategy have been detailed in previous publications^13^. In brief, a random cluster sample (age range 20 to 79 years) was drawn from the population of West Pomerania, the north-eastern region of Germany. The net sample (without migrated or deceased persons) comprised 6265 eligible individuals with 4308 (2193 women) of them participating in the baseline (SHIP-START-0) study (response rate 68.8%). All subjects who participated in the baseline SHIP were re-invited to take part in the first examination follow-up (SHIP-START-1), which was realized from 2002 to 2006. Of the 3949 persons eligible for SHIP-START-1, 3300 subjects were re-examined, resulting in a follow-up response of 83.6%. For the second examination follow-up (SHIP-START-2) conducted from 2008 to 2012, all 3708 eligible individuals that participated in the baseline study were re-invited. Of them, 2333 were re-examined (follow-up response of 62.9%).

While SHIP-START-2 was being conducted, between 2008 and 2012, a second independent cohort was established, called SHIP-TREND-0, covering the same region as the initial SHIP. A stratified random sample of 8826 adults, aged 20 to 79 years, was selected. Participation in the initial SHIP-START cohort was an exclusion criterion. In total, 4420 individuals participated in SHIP-TREND-0 (response rate 50.1%). For the present study, we performed cross-sectional analyses using pooled data from SHIP-START-2 and SHIP-TREND-0 (n = 6753 individuals; 3510 women [52.0%]). We excluded participants with previous self-reported heart failure or dyspnoea (equivalent to NYHA class ≥ II) combined with NT-proBNP ≥ 125 ng/l (n = 656), LVEF, determined by echocardiography, lower or equal than 40% (n = 4). We also excluded individuals with missing values for echocardiography (n = 1427), blood pressure (n = 22) or any of the covariables (n = 36). The final analytical sample comprised 4608 subjects (2454 women; 53.3%) aged 20 to 90 years (Supplementary Fig. 1). All study participants gave written informed consent. The study was approved by the ethics committee of the University of Greifswald and complies with the Declaration of Helsinki.

Conducted in Mid-Western Germany between April 2012 and April 2017, GHS is a large population-based, prospective, single-centre cohort study. A description of the study design was published before^14^. Participants underwent a standardized investigational plan including assessment of cardiovascular risk factors and comorbidities, clinical examinations, and laboratory analysis of venous blood samples. From the 15,010 cohort, 14,963 subjects received an echocardiography. From this cohort, subjects with self-reported heart failure or dyspnoea (NYHA class ≥ II) combined with NT-proBNP ≥ 125 ng/l (n = 198) or LVEF ≤ 40% (n = 47) or missing values in data on systolic or diastolic blood pressure (n = 16) were excluded. Finally, 14,761 subjects remained for subsequent analyses. The local ethics committee and data protection officer approved the study protocol before initiation. Written informed consent was provided by all study participants. Study procedures were conducted in line with the principles outlined in the Declaration of Helsinki.

### Statistical analysis

Continuous variables are presented as median and interquartile range, categorical variables are presented as absolute numbers and percentages. Comparisons between subgroups were performed using the Mann–Whitney-U-Test for continuous variables and the Fisher test for categorical variables. In order to examine the association of systolic blood pressure, diastolic blood pressure, and pulse pressure with various parameters, a multiple linear regression model was calculated with blood pressure as a predictor and the respective echocardiographic parameters as outcome. A separate model was created for each outcome and BP. Adjustment was performed for age, sex, body mass index (BMI), smoking, coronary artery disease, diabetes, antihypertensive medication, and LV mass. The numerical predictors were centered and scaled, i.e. each divided by their standard deviation after subtracting the mean, so that the result of the estimation parameter beta can be interpreted in terms of standard deviation. A p-value of < 0.05 was considered as statistically significant. All tests were two tailed. Data analysis was performed using R version 4.0.3. Each cohort data set was analysed independently.

## Results

### Baseline characteristics

7396 subjects of the first 10,000 HCHS participants qualified for the analysis after applying the exclusion criteria (Fig. 1). Validation cohorts comprised 4608 subjects from the Study of Health in Pomerania (SHIP) and 14,715 subjects from the Gutenberg Health Study (GHS). The HCHS cohort showed the expected characteristics of a middle-aged European population, with 3830 (51.8%) women, a median age of 62 [IQR: 55; 69] years and a median BMI of 26.0 [IQR: 23.4; 29.0] kg/m^2^ (Table 1).

**Table 1:** Baseline characteristics of the HCHS derivation study population.

	Blood pressure		p-value
	≥ 140/90 mmHg	< 140/90 mmHg	
n (%)	3478 (47.0)	3918 (53.0)	
Demographics and biological data			
Age, years	65 [58, 71]	60 [53, 67]	< 0.001
Female	1563 (44.9)	2267 (57.9)	< 0.001
BMI, kg/m^2^	26.6 [24.1, 29.6]	25.4 [23.0, 28.5]	< 0.001
Current smoking	591 (17.1)	852 (21.8)	< 0.001
Dyspnoea	170 (5.4)	210 (6.0)	0.315
Systolic blood pressure, mmHg	151.5 [144.0, 161.5]	126.0 [118.5, 132.5]	< 0.001
Diastolic blood pressure, mmHg	88.0 [82.5, 94.0]	77.5 [72.0, 82.0]	< 0.001
Comorbidities			
Diabetes	301 (9.3)	224 (6.2)	< 0.001
Coronary artery disease	183 (5.7)	155 (4.3)	0.006
Atrial fibrillation	168 (5.4)	143 (4.1)	0.012
Asthma or COPD	175 (5.5)	216 (6.0)	0.415
Peripheral artery disease	83 (2.6)	107 (3.0)	0.405
Medication			
Aldosterone antagonists	14 (0.4)	17 (0.5)	0.972
Loop diuretics	53 (1.6)	47 (1.3)	0.275
Thiazide diuretics	94 (2.8)	66 (1.8)	0.004
Betablocker	653 (19.6)	442 (11.8)	< 0.001
ACEi/ARBs	1175 (35.3)	840 (22.5)	< 0.001
Calcium channel blockers	314 (9.4)	216 (5.8)	< 0.001
Laboratories			
GFR, ml/min	84.1 [73.6, 92.4]	87.7 [77.2, 95.6]	< 0.001
NT-proBNP, ng/l	85.0 [47.0, 156.0]	71.0 [40.0, 120.0]	< 0.001
Hemoglobin, g/dl	14.5 [13.8, 15.3]	14.1 [13.4, 14.9]	< 0.001
HbA1c, %	5.6 [5.3, 5.8]	5.5 [5.3, 5.7]	< 0.001

Continuous variables are presented as median and interquartile range, and categorical variables are presented as absolute numbers and percentages.

*ACEi* angiotensin-converting enzyme inhibitor, *ARB* angiotensin receptor blocker, *BMI* body mass index, *BP* blood pressure, *GFR* glomerular filtration rate, *NT-proBNP* N-terminal pro-B-type natriuretic peptide.

Median systolic blood pressure was 137.0 [IQR: 125.5, 150.5] millimeters of mercury (mmHg) and median diastolic blood pressure 82.0 [75.5, 88.0] mmHg. 3478 (47%) had a systolic blood pressure of ≥ 140 mmHg, a diastolic blood pressure of ≥ 90 mmHg or both (high blood pressure). Those with high blood pressure were older, had a higher BMI as well as a higher burden of diabetes and atrial fibrillation than those with normal blood pressure.

Baseline left atrial and ventricular echocardiographic parameters including LV mass, LVEF, E/E′, and volumes are shown in Table 2, ranges of LVEF are displayed in Supplementary Table 1. At least one indicator of diastolic dysfunction namely E/E′ > 14, left atrial volume index > 34 ml/m^2^ or maximal velocity of tricuspid regurgitation (TR Vmax) > 2.8 m/s was present in 1013 (14.5%) participants. Six (0.1%) participants fulfilled all three criteria of diastolic dysfunction all of which had a blood pressure of ≥ 140 mmHg/90 mmHg.

**Table 2:** Echocardiographic characteristics of subjects with and without arterial hypertension from HCHS.

	Blood pressure		p-value
	≥ 140/90 mmHg	< 140/90 mmHg	
Functional parameters			
LVEF, %	58.2 [55.3, 61.6]	58.8 [56.0, 62.1]	< 0.001
E/e′ mean ratio	7.6 [6.4, 9.0]	7.0 [6.0, 8.3]	< 0.001
LV lateral e′, cm/s	9.6 [8.0, 11.5]	10.8 [8.9, 12.9]	< 0.001
LV septal e′, cm/s	7.9 [6.7, 9.5]	8.9 [7.4, 10.6]	< 0.001
LAVI, ml/m^2^	25.7 [20.7, 31.9]	24.6 [19.9, 29.6]	< 0.001
Morphological parameters			
LVMI, g/m^2.7^	38.6 [32.8, 45.5]	34.6 [29.8, 40.5]	< 0.001
IVSD, mm	10.2 [9.1, 11.4]	9.5 [8.5, 10.6]	< 0.001
RWT, mm	0.409 [0.367, 0.459]	0.388 [0.350, 0.433]	< 0.001
LVPWD, mm	9.4 [8.5, 10.4]	8.8 [8.0, 9.7]	< 0.001
LVEDD, mm	47.9 [44.4, 51.4]	47.3 [43.9, 50.5]	< 0.001
LVEDV, ml	111.3 [93.0, 134.3]	107.8 [90.8, 130.1]	< 0.001

*IVSD* interventricular septum diameter, *LA* left atrial, *LAVI* left atrial systolic volume indexed to body surface area, *LVEDD* left ventricular end-diastolic diameter, *LV*  left ventricle, *LVEDV* left ventricular end-diastolic volume, *LVEF* left ventricular ejection fraction, *LVMI* left ventricular mass indexed to height^2.7^, *RWT* relative wall thickness.

### Left ventricular mass and wall thickness

**Figure 2:**
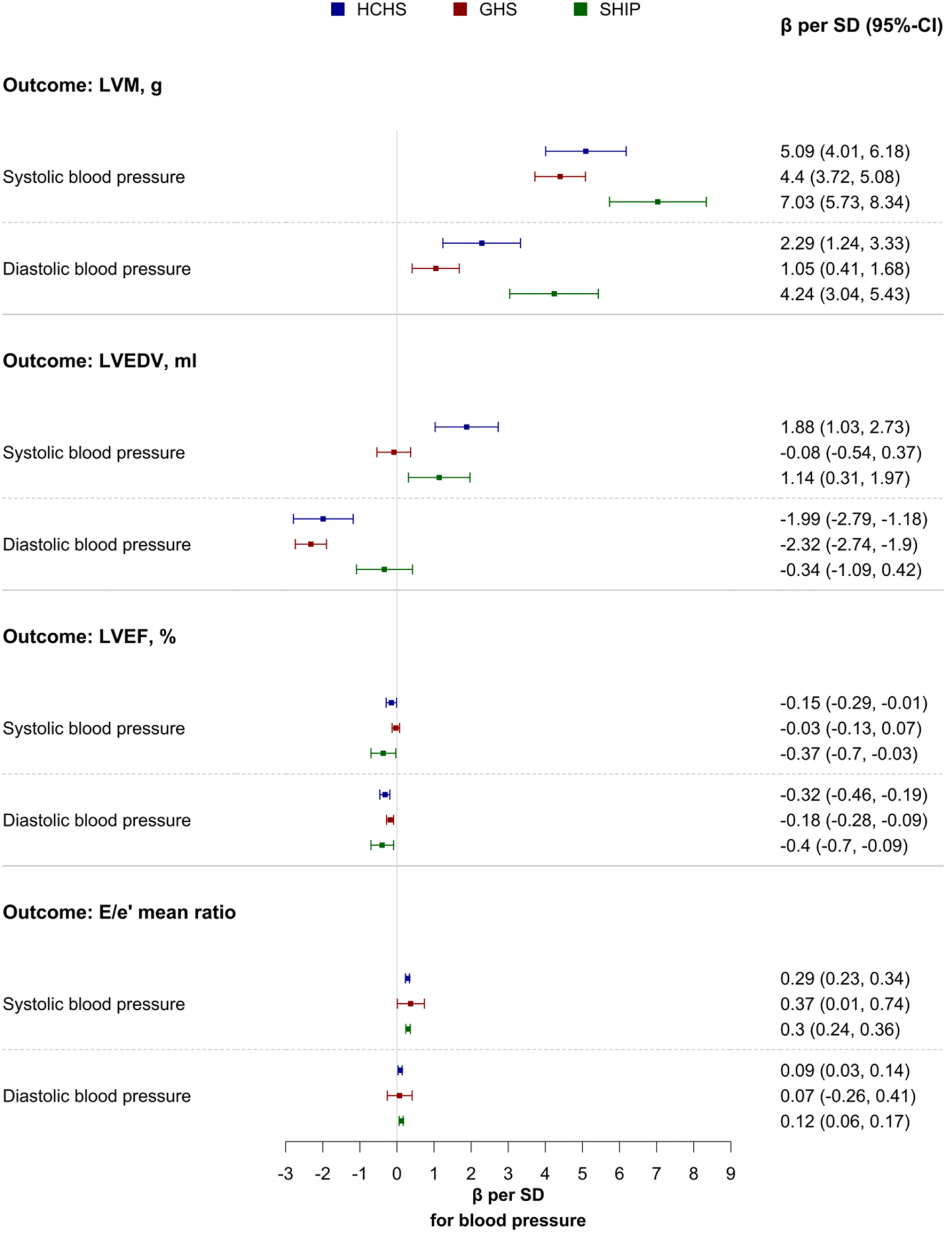
Forest plot of multivariable linear regression analysis of systolic and diastolic blood pressure as predictor and LV measures as outcome in subjects without heart failure from the Hamburg City Health Study (blue), Gutenberg Health Study (red) and the Study of Health in Pomerania (green). Adjustment was performed for age, sex, BMI, smoking, coronary artery disease (prevalent myocardial infarction in GHS), diabetes, atrial fibrillation, and antihypertensive medication. *SD* standard deviation, *CI* confidence interval, *LV* left ventricular, *LVM* left ventricular mass, *LVEDV* left ventricular end-diastolic volume, *LVEF* LV ejection fraction.

In multivariable linear regression analysis adjusted for common cardiovascular risk markers, continuous blood pressure measures were strongly associated with indicators of left ventricular hypertrophy in all cohorts (Table 3). Systolic blood pressure and diastolic blood pressure correlated with left ventricular mass with a β per SD (in g) of 5.09 (95% CI 4.01; 6.18, p < 0.001) and 2.29 (95% CI 1.24; 3.33, p < 0.001), respectively in HCHS (Fig. 2, Table 2, Supplementary Tables 2 and 3). Concordantly, systolic blood pressure and diastolic blood pressure demonstrated associations with relative wall thickness in HCHS, SHIP, and GHS (β per SD from HCHS (in mm) for systolic blood pressure: 0.0075, 95% CI 0.0053; 0.0097, p < 0.001; β per SD from HCHS for diastolic blood pressure: 0.0099, 95% CI 0.0079; 0.012, p < 0.001). In addition, not only systolic blood pressure and diastolic blood pressure but also pulse pressure demonstrated associations with parameters of left ventricular hypertrophy.

**Table 3:** Multivariable univariate linear regression analysis for the association of systolic and diastolic blood pressure with echocardiographic variables from HCHS.

	Systolic blood pressure		Diastolic blood pressure		Pulse Pressure	
	ß per SD (95% CI)	p-value	ß per SD (95% CI)	p-value	ß per SD (95% CI)	p-value
Functional parameters						
LVEF, %	− 0.15 [− 0.29; − 0.01]	0.041	− 0.32 [− 0.46; − 0.19]	< 0.001	0.05 [− 0.1; 0.2]	0.516
E/e*′* mean ratio	0.29 [0.23; 0.34]	< 0.001	0.09 [0.03; 0.14]	0.001	0.34 [0.28; 0.4]	< 0.001
LV lateral e*′*, cm/s	− 0.27 [− 0.35; − 0.19]	< 0.001	− 0.47 [− 0.54; − 0.4]	< 0.001	− 0.01 [− 0.09; 0.07]	0.876
LV septal e*′*, cm/s	− 0.26 [− 0.32; − 0.19]	< 0.001	− 0.35 [− 0.41; − 0.28]	< 0.001	− 0.09 [− 0.16; − 0.02]	0.016
LASV, ml	0.69 [0.42; 0.95]	< 0.001	− 0.3 [− 0.55; − 0.05]	0.018	1.22 [0.95; 1.49]	< 0.001
Morphological parameters						
LVM, g	5.09 [4.01; 6.18]	< 0.001	2.29 [1.24; 3.33]	< 0.001	5.42 [4.28; 6.55]	< 0.001
IVSD, mm	0.21 [0.16; 0.25]	< 0.001	0.21 [0.17; 0.25]	< 0.001	0.12 [0.08; 0.17]	< 0.001
RWT, mm	0.00748 [0.00532; 0.00964]	< 0.001	0.00988 [0.00783; 0.01193]	< 0.001	0.00264 [0.00037; 0.0049]	0.022
LVPWD, mm	0.18 [0.15; 0.22]	< 0.001	0.14 [0.1; 0.17]	< 0.001	0.15 [0.11; 0.19]	< 0.001
LVEDD, mm	0.12 [− 0.01; 0.25]	0.075	− 0.26 [− 0.38; − 0.13]	< 0.001	0.38 [0.24; 0.52]	< 0.001
LVEDV, ml	1.88 [1.03; 2.73]	< 0.001	− 1.99 [− 2.79; − 1.18]	< 0.001	4.29 [3.41; 5.16]	< 0.001

Adjustment was performed for age, sex, BMI, smoking, coronary artery disease, diabetes, atrial fibrillation, and antihypertensive medication. Abbreviations as in Table 2.

### Systolic and diastolic function

Diastolic blood pressure was associated with declining systolic function in all cohorts with a β per SD (in %) of -0.32 (95% CI − 0.46; − 0.19 p < 0.001) for LVEF in HCHS. Systolic blood pressure was associated with declining systolic function as indicated by increasing E/E′ (β per SD for systolic blood pressure in HCHS: 0.29, 95% CI 0.23; 0.34, p < 0.001) in HCHS, GHS, and SHIP.

### Left atrial and ventricular volumes

Higher systolic blood pressure was associated with increased left atrial volume in HCHS (β per SD in ml for systolic blood pressure and left atrial systolic volume: 0.69, 95% CI 0.42; 0.95, p < 0.001) and SHIP as indicated by associations with left atrial end-diastolic diameter (LAEDD). Data on left atrial volume in GHS were not available.

Higher diastolic blood pressure was associated with reduced left ventricular end-diastolic volume in HCHS and GHS as demonstrated by a β per SD (in ml) of − 1.99 (95% CI − 2.79; − 1.18, p < 0.001) in HCHS. The same trend, but lacking statistical significance, was detectable in SHIP (β per SD for: − 0.34, 95% CI − 1.09; 0.42, p = 0.383).

### Pulse pressure and left ventricular measures

Pulse pressure showed associations with left ventricular mass and wall thickness and declining diastolic function but not with declining systolic function throughout all cohorts (Table 3 and Supplementary Table 2).

### Sensitivity analyses in HCHS

The described associations of systolic blood pressure and diastolic blood pressure with morphological and functional measures persisted after additional adjustment for left ventricular mass (Supplementary Table 7) in HCHS. Furthermore, our key findings were consistent in sensitivity analyses excluding those participants taking antihypertensive medication as well as in men and women separately in HCHS (Supplementary Tables 4, 5, 6).

## Discussion

This study in almost 27,000 individuals from three independent population-based cohorts free of heart failure symptoms found that both systolic and diastolic blood pressure are related to increased left ventricular mass. Elevated systolic blood pressure was associated with declining diastolic function, while elevated diastolic blood pressure was associated with declining left ventricular systolic function robustly across all cohorts. These findings provide a detailed picture of the interplay of elevated systolic and diastolic blood pressure with cardiac structure and function. Our findings might suggest that future clinical studies may test differential therapeutic approaches to elevated systolic and diastolic blood pressure in the prevention of heart failure.

### Blood pressure and its correlation with left ventricular hypertrophy and diastolic dysfunction

The fact that 45.8% of HCHS participants presented with a systolic blood pressure of ≥ 140 mmHg or diastolic blood pressure ≥ 90 mmHg or both stresses the incremental burden of arterial hypertension in the middle-aged Western population. Arterial hypertension induces left ventricular wall stress resulting in left ventricular hypertrophy^17^. Consequently and in line with most of the so far published studies, systolic blood pressure, diastolic blood pressure, and pulse pressure were strongly associated with left ventricular hypertrophy in our asymptomatic study population throughout all cohorts^18^. Especially elevated systolic blood pressure is an established and important risk factor not only for pathological reverse cardiac remodelling but also for newly diagnosed heart failure^19–21^. Our data support this by showing that elevated systolic blood pressure and elevated pulse pressure are associated with declining diastolic function indicated by elevated E/E′ and atrial enlargement. Although left ventricular hypertrophy is considered a major driver of diastolic dysfunction, data from the Third Generation cohort of the Framingham Heart Study suggests correlations of blood pressure with diastolic dysfunction independently of left ventricular hypertrophy^18^. In line with those results, correlations of systolic blood pressure and pulse pressure with declining diastolic function remained significant even after adjustment for left ventricular mass in HCHS (Supplementary Table 4). Changes of the left ventricular relaxation process during diastole might actually occur already before the echocardiographic manifestation of left ventricular hypertrophy due to structural alterations of myocardial tissue composition at the cellular level^22^. Diastolic blood pressure on the other hand was not associated with declining diastolic function. Whereas systolic blood pressure is closely related to increased afterload induced by stiffened arteries, diastolic blood pressure reflects the pressure during relaxation and is less related to mechanical cardiac demands. Elevated systolic blood pressure therefore induces a greater amount of cardiac remodelling compared to diastolic blood pressure. Accordingly, we found a higher association of systolic blood pressure than diastolic blood pressure with left ventricular hypertrophy.

### Blood pressure and left ventricular systolic dysfunction

Elevated diastolic blood pressure, but not elevated systolic blood pressure, was robustly associated with declining left ventricular systolic function in this analysis comprising three independent cohorts. Pathophysiologically, blood pressure induced systolic dysfunction might be best explained by disturbed ventricular-arterial coupling: high diastolic blood even more than systolic blood pressure is associated with arterial stiffness^23^. This may lead to a reduction in coronary perfusion pressure inducing myocardial ischaemia and deteriorating left ventricular systolic function. Interestingly, not only high but also low diastolic blood pressure has previously been found to be related to heart failure^24^. However, the effect of elevated systolic blood pressure compared to diastolic blood pressure on the development of heart failure was shown to be much stronger^20^. Detrimental effects of diastolic blood pressure on systolic function might partially be counterbalanced by beneficial effects including left ventricular volume reduction, as demonstrated by a trend in our study.

Accordingly, conflicting data on the effect of diastolic hypertension on cardiovascular outcome were reported before: Flint et al. report increased risk of adverse cardiovascular outcome whereas data from the Atherosclerosis Risk in Communities (ARIC) Study found no association^20, 25^. Unexpectedly, elevated systolic blood pressure was not consistently linked to systolic dysfunction in our study. Although we excluded patients with heart failure from our analysis, we cannot exclude residual confounding of lower systolic blood pressures due to heart failure which may partially explain the lack of association between heart failure and elevated systolic blood pressure in this cross-sectional analysis. Nevertheless, our findings clearly highlight associations of systolic blood pressure with declining diastolic function and diastolic blood pressure with declining systolic function.

### Limitations

Participants of HCHS as well as from the validation cohorts SHIP and GHS were mainly of Caucasian descent. Accordingly, our findings cannot be transferred to other ethnic groups.

The definition of heart failure comprised self-reported heart failure, dyspnoea equivalent to NYHA II-III, and an NT-proBNP concentration of more than 125 pg/ml and LVEF ≤ 40%. Heart failure might thus be underreported. Blood pressure was measured by medical professionals at the day of the baseline visit, which might have led to falsely high values. Additionally, the intake of antihypertensive medication might considerably influence the reported results. In order to reduce the risk of relevant confounding we have adjusted for antihypertensive medication and performed a sensitivity analysis excluding those taking antihypertensive medication (Supplemental Table 4). Finally, the cross-sectional nature of our study only allows the description of associations; no causal claims can be drawn, which is particularly relevant as some of the described associations are possibly bidirectional.

## Conclusions

In this large community-based study of three German adult populations, both elevated systolic and diastolic blood pressure were associated with left ventricular hypertrophy. Only systolic blood pressure was related to declining diastolic function whereas diastolic blood pressure was associated with declining left ventricular systolic function. These results suggest a differential role of systolic blood pressure and diastolic blood pressure for the development of heart failure.

### Supplementary Information

Supplementary Information.

## Data Availability

The data underlying this article cannot be shared publicly due to the privacy of individuals that participated in the study. The data will be shared on reasonable request to the corresponding author.

## Abbreviations

AF: Atrial fibrillation
ARB: Angiotensin receptor blocker
BMI: Body mass index
BP: Blood pressure
COPD: Chronic obstructive pulmonary disease
GFR: Glomerular filtration rate
EACVI: European Association of Cardiovascular Imaging
ECG: Electrocardiogram
ESC: European Society of Cardiology
GHS: Gutenberg Health Study
HCHS: Hamburg City Health Study
HF: Heart failure
HFpEF: Heart failure with preserved ejection fraction
LAVI: Left atrial systolic volume indexed to body surface area
LV: Left ventricle/ventricular
LAEDD: Left atrial enddiastolic diameter
LVEDD: Left ventricular enddiastolic diameter
LVEF: Left ventricular ejection fraction
LVMI: Left ventricular mass index
NT-proBNP: N-terminal pro-brain natriuretic peptide
PP: Pulse pressure
RV: Right ventricle/ventricular
SHIP: Study of Health in Pomerania
TR Vmax: Maximal tricuspid regurgitation velocity
TTE: Transthoracic echocardiography
