# Supplementary material for: Correlation of systolic and diastolic blood pressure with echocardiographic phenotypes of cardiac structure and function from three German population-based studies
Source: Sci Rep. 2023 Sep 4;13:14525. doi: 10.1038/s41598-023-41571-x (PMC10477248; doi:10.1038/s41598-023-41571-x)
Supplement: Supplementary file 1 — Supplementary Information. [file 41598_2023_41571_MOESM1_ESM.docx]

**Supplementary**

| LVEF Range | Overall | >=140/90 mmHg | | <140/90 mmHg |
| --- | --- | --- | --- | --- |
|  | | | | |
| n | 7396 | | 3478 | 3918 |
| >40% - <50% | 249 (3.7) | | 135 (4.3) | 114 (3.2) |
| >=50% - <=55% | 1094 (16.1) | | 581 (18.4) | 513 (14.2) |
| >55% - <60% | 2863 (42.3) | | 1306 (41.3) | 1557 (43.1) |
| >=60% | 2569 (37.9) | | 1139 (36.0) | 1430 (39.6) |

**Supplementary Table 1: Range of left ventricular ejection fraction.** The number and percentage of participants is stratified according to left ventricular ejection fraction ranges and blood pressure. *Abbreviations: LVEF = left ventricular ejection fraction.*

|  | Systolic blood pressure | | Diastolic blood pressure | | Pulse Pressure | |
| --- | --- | --- | --- | --- | --- | --- |
|  | **ß per SD (95% CI)** | **p-value** | **ß per SD (95% CI)** | **p-value** | **ß per SD (95% CI)** | **p-value** |
| **FUNCTIONAL PARAMETERS** | | | | | | |
| LVEF, % | -0.37 [-0.70; -0.03] | 0.031 | -0.40 [-0.70; -0.09] | 0.010 | -0.13 [-0.46; 0.19] | 0.429 |
| E/e' mean ratio | 0.30 [0.24; 0.36] | <0.001 | 0.12 [0.06; 0.17] | <0.001 | 0.29 [0.24; 0.35] | <0.001 |
| LV lateral e', cm/s | 0.31 [-0.02; 0.08] | 0.210 | -0.04 [-0.08; 0.00] | 0.073 | 0.08 [0.03; 0.12] | 0.001 |
| LV septal e’, cm/s | 0.39 [0.32; 0.46] | <0.001 | 0.20 [0.14; 0.27] | <0.001 | 0.34 [0.26; 0.41] | <0.001 |
| **MORPHOLOGICAL PARAMETERS** | | | | | | |
| LVM, g | 7.03 [5.73; 8.34] | <0.001 | 4.24 [3.04; 5.43] | <0.001 | 5.58 [4.29; 6.86] | <0.001 |
| IVSD, mm | 0.22 [0.13; 0.31] | <0.001 | 0.16 [0.08; 0.24] | <0.001 | 0.15 [0.07; 0.24] | <0.001 |
| RWT, mm | 0.00814 [0.00522; 0.01108] | <0.001 | 0.00853 [0.00588; 0.01118] | <0.001 | 0.00318 [0.00030; 0.00605] | 0.030 |
| LVEDD, mm | 0.21 [0.06; 0.36] | 0.006 | -0.07 [-0.21; 0.07] | 0.333 | 0.34 [0.20; 0.49] | <0.001 |
| LVEDV, ml | 1.14 [0.31; 1.97] | 0.007 | -0.34 [-1.09; 0.42] | 0.383 | 1.83 [1.02; 2.64] | <0.001 |
| LAEDD, mm | 0.33 [0.17; 0.48] | <0.001 | 0.06 [-0.08; 0.20] | 0.381 | 0.38 [0.23; 0.53] | <0.001 |

**Supplementary Table 2: Multivariable linear regression analysis for the association of systolic and diastolic blood pressure with echocardiographic variables from SHIP** (n = 4,608)**.** *Linear regression model adjusted for age, sex, body mass index, smoking, prevalent myocardial infarction, prevalent diabetes mellitus, prevalent atrial fibrillation and use of antihypertensive medication.

|  | Systolic blood pressure | | Diastolic blood pressure | | Pulse Pressure | |
| --- | --- | --- | --- | --- | --- | --- |
|  | **ß per SD (95% CI)** | **p-value** | **ß per SD (95% CI)** | **p-value** | **ß per SD (95% CI)** | **p-value** |
| **FUNCTIONAL PARAMETERS** | | | | | | |
| LVEF, % | -0.03 [ -0.13 ; 0.07 ] | 0.536 | -0.18 [ -0.28 ; -0.09 ] | <0.001 | 0.13 [ 0.02 ; 0.23 ] | 0.021 |
| E/e' mean ratio | 0.37 [ 0.01 ; 0.74 ] | 0.044 | 0.07 [ -0.26 ; 0.41 ] | 0.663 | 0.48 [ 0.1 ; 0.86 ] | 0.013 |
| LV lateral e', cm/s | -0.43 [ -0.51 ; -0.35 ] | <0.001 | -0.56 [ -0.64 ; -0.48 ] | <0.001 | -0.13 [ -0.22 ; -0.04 ] | 0.004 |
| **MORPHOLOGICAL PARAMETERS** | | | | | | |
| LVM, g | 4.4 [ 3.72 ; 5.08 ] | <0.001 | 1.05 [ 0.41 ; 1.68 ] | 0.001 | 5.54 [ 4.82 ; 6.25 ] | <0.001 |
| IVSD, mm | 0.28 [ 0.25 ; 0.31 ] | <0.001 | 0.23 [ 0.21 ; 0.26 ] | <0.001 | 0.19 [ 0.16 ; 0.22 ] | <0.001 |
| RWT, mm | 0.01191 [ 0.01043 ; 0.01339 ] | <0.001 | 0.01302 [ 0.01165 ; 0.01438 ] | <0.001 | 0.00541 [ 0.00385 ; 0.00698 ] | <0.001 |
| LVPWD, mm | 0.2 [ 0.17 ; 0.23 ] | <0.001 | 0.14 [ 0.11 ; 0.17 ] | <0.001 | 0.16 [ 0.13 ; 0.19 ] | <0.001 |
| LVEDD, mm | -0.17 [ -0.25 ; -0.1 ] | <0.001 | -0.37 [ -0.44 ; -0.3 ] | <0.001 | 0.09 [ 0.01 ; 0.17 ] | 0.03 |
| LVEDV, ml | -0.08 [ -0.54 ; 0.37 ] | 0.718 | -2.32 [ -2.74 ; -1.9 ] | <0.001 | 2.06 [ 1.59 ; 2.53 ] | <0.001 |

**Supplementary Table 3: Multivariable linear regression analysis for the association of systolic and diastolic blood pressure with echocardiographic variables from GHS.** Adjustment was performed for age, sex, BMI, smoking, coronary artery disease, diabetes, atrial fibrillation, and antihypertensive medication. Dotted lines represent the 95%-confidence intervals. *Abbreviations: BP = blood pressure, LV = left ventricular, LVEDV = left ventricular end-diastolic volume.*

|  | Systolic blood pressure | | Diastolic blood pressure | | Pulse Pressure | |
| --- | --- | --- | --- | --- | --- | --- |
|  | **ß per SD (95% CI)** | **p-value** | **ß per SD (95% CI)** | **p-value** | **ß per SD (95% CI)** | **p-value** |
| **FUNCTIONAL PARAMETERS** | | | | | | |
| LVEF, % | -0.22 [ -0.42 ; -0.02 ] | 0.031 | -0.37 [ -0.56 ; -0.19 ] | <0.001 | -0.02 [ -0.23 ; 0.2 ] | 0.882 |
| E/e' mean ratio | 0.28 [ 0.2 ; 0.36 ] | <0.001 | 0.08 [ 0 ; 0.15 ] | 0.037 | 0.34 [ 0.25 ; 0.42 ] | <0.001 |
| LV lateral e', cm/s | -0.21 [ -0.31 ; -0.1 ] | <0.001 | -0.42 [ -0.52 ; -0.33 ] | <0.001 | 0.05 [ -0.06 ; 0.16 ] | 0.4 |
| LV septal e’, cm/s | -0.22 [ -0.32 ; -0.12 ] | <0.001 | -0.35 [ -0.44 ; -0.26 ] | <0.001 | -0.04 [ -0.14 ; 0.07 ] | 0.48 |
| LASV, ml | 0.67 [ 0.33 ; 1 ] | <0.001 | -0.15 [ -0.47 ; 0.17 ] | 0.361 | 1.07 [ 0.72 ; 1.43 ] | <0.001 |
| **MORPHOLOGICAL PARAMETERS** | | | | | | |
| LVM, g | 4.05 [ 2.82 ; 5.29 ] | <0.001 | 2.56 [ 1.41 ; 3.72 ] | <0.001 | 3.69 [ 2.4 ; 4.98 ] | <0.001 |
| IVSD, mm | 0.17 [ 0.11 ; 0.22 ] | <0.001 | 0.2 [ 0.14 ; 0.25 ] | <0.001 | 0.08 [ 0.02 ; 0.14 ] | 0.01 |
| RWT, mm | 0.00547 [ 0.00245 ; 0.00848 ] | <0.001 | 0.00913 [ 0.00634 ; 0.01192 ] | <0.001 | 0.00039 [ -0.00277 ; 0.00354 ] | 0.809 |
| LVPWD, mm | 0.13 [ 0.08 ; 0.18 ] | <0.001 | 0.13 [ 0.08 ; 0.17 ] | <0.001 | 0.09 [ 0.03 ; 0.14 ] | 0.001 |
| LVEDD, mm | 0.17 [ -0.01 ; 0.35 ] | 0.058 | -0.15 [ -0.31 ; 0.02 ] | 0.076 | 0.36 [ 0.18 ; 0.55 ] | <0.001 |
| LVEDV, ml | 1.58 [ 0.58 ; 2.58 ] | 0.002 | -1.3 [ -2.25 ; -0.36 ] | 0.007 | 3.29 [ 2.25 ; 4.33 ] | <0.001 |

**Supplementary Table 4. Multivariable linear regression analysis for the association of systolic and diastolic blood pressure with echocardiographic variables from HCHS only females.** Adjustment was performed for age, sex, BMI, smoking, coronary artery disease, diabetes, atrial fibrillation, and antihypertensive medication. Abbreviations as in *Table 2*.

**Supplementary Table 5. Multivariable linear regression analysis for the association of systolic and diastolic blood pressure with echocardiographic variables from HCHS only males.** Adjustment was performed for age, sex, BMI, smoking, coronary artery disease, diabetes, atrial fibrillation, and antihypertensive medication. Abbreviations as in *Table 2*.

|  | Systolic blood pressure | | Diastolic blood pressure | | Pulse Pressure | |
| --- | --- | --- | --- | --- | --- | --- |
|  | **ß per SD (95% CI)** | **p-value** | **ß per SD (95% CI)** | **p-value** | **ß per SD (95% CI)** | **p-value** |
| **FUNCTIONAL PARAMETERS** | | | | | | |
| LVEF, % | -0.08 [ -0.28 ; 0.12 ] | 0.422 | -0.28 [ -0.47 ; -0.08 ] | 0.005 | 0.11 [ -0.1 ; 0.32 ] | 0.293 |
| E/e' mean ratio | 0.28 [ 0.2 ; 0.35 ] | <0.001 | 0.08 [ 0.01 ; 0.16 ] | 0.028 | 0.33 [ 0.25 ; 0.41 ] | <0.001 |
| LV lateral e', cm/s | -0.3 [ -0.41 ; -0.2 ] | <0.001 | -0.49 [ -0.59 ; -0.39 ] | <0.001 | -0.03 [ -0.14 ; 0.08 ] | 0.606 |
| LV septal e’, cm/s | -0.26 [ -0.35 ; -0.17 ] | <0.001 | -0.32 [ -0.41 ; -0.23 ] | <0.001 | -0.11 [ -0.21 ; -0.02 ] | 0.022 |
| LASV, ml | 0.78 [ 0.39 ; 1.18 ] | <0.001 | -0.38 [ -0.76 ; 0 ] | 0.048 | 1.42 [ 1.02 ; 1.82 ] | <0.001 |
| **MORPHOLOGICAL PARAMETERS** | | | | | | |
| LVM, g | 6.32 [ 4.58 ; 8.06 ] | <0.001 | 2.28 [ 0.56 ; 4.01 ] | 0.009 | 7.25 [ 5.42 ; 9.08 ] | <0.001 |
| IVSD, mm | 0.24 [ 0.18 ; 0.3 ] | <0.001 | 0.22 [ 0.16 ; 0.28 ] | <0.001 | 0.16 [ 0.1 ; 0.23 ] | <0.001 |
| RWT, mm | 0.00891 [ 0.00588 ; 0.01195 ] | <0.001 | 0.01028 [ 0.00731 ; 0.01325 ] | <0.001 | 0.00442 [ 0.00121 ; 0.00763 ] | 0.007 |
| LVPWD, mm | 0.23 [ 0.17 ; 0.29 ] | <0.001 | 0.15 [ 0.09 ; 0.2 ] | <0.001 | 0.21 [ 0.15 ; 0.27 ] | <0.001 |
| LVEDD, mm | 0.1 [ -0.1 ; 0.3 ] | 0.326 | -0.34 [ -0.53 ; -0.15 ] | 0.001 | 0.42 [ 0.21 ; 0.63 ] | <0.001 |
| LVEDV, ml | 2.36 [ 1 ; 3.72 ] | 0.001 | -2.56 [ -3.87 ; -1.24 ] | <0.001 | 5.48 [ 4.08 ; 6.88 ] | <0.001 |

**Supplementary Table 6. Multivariable linear regression analysis for the association of systolic and diastolic blood pressure with echocardiographic variables from HCHS, only subjects without antihypertensive medication.** Adjustment was performed for age, sex, BMI, smoking, coronary artery disease, diabetes and atrial fibrillation. Abbreviations as in *Table 2*.

|  | Systolic blood pressure | | Diastolic blood pressure | | Pulse Pressure | |
| --- | --- | --- | --- | --- | --- | --- |
|  | **ß per SD (95% CI)** | **p-value** | **ß per SD (95% CI)** | **p-value** | **ß per SD (95% CI)** | **p-value** |
| **FUNCTIONAL PARAMETERS** | | | | | | |
| LVEF, % | -0.26 [ -0.44 ; -0.09 ] | 0.003 | -0.39 [ -0.56 ; -0.22 ] | <0.001 | -0.06 [ -0.24 ; 0.12 ] | 0.517 |
| E/e' mean ratio | 0.3 [ 0.23 ; 0.36 ] | <0.001 | 0.12 [ 0.06 ; 0.18 ] | <0.001 | 0.33 [ 0.27 ; 0.39 ] | <0.001 |
| LV lateral e', cm/s | -0.3 [ -0.4 ; -0.21 ] | <0.001 | -0.49 [ -0.58 ; -0.4 ] | <0.001 | -0.03 [ -0.13 ; 0.06 ] | 0.525 |
| LV septal e’, cm/s | -0.24 [ -0.32 ; -0.15 ] | <0.001 | -0.31 [ -0.4 ; -0.23 ] | <0.001 | -0.09 [ -0.18 ; 0 ] | 0.045 |
| LASV, ml | 0.52 [ 0.21 ; 0.83 ] | 0.001 | -0.22 [ -0.51 ; 0.08 ] | 0.149 | 0.93 [ 0.62 ; 1.25 ] | <0.001 |
| **MORPHOLOGICAL PARAMETERS** | | | | | | |
| LVM, g | 5.27 [ 4 ; 6.53 ] | <0.001 | 2.99 [ 1.76 ; 4.21 ] | <0.001 | 5.08 [ 3.8 ; 6.37 ] | <0.001 |
| IVSD, mm | 0.23 [ 0.18 ; 0.28 ] | <0.001 | 0.24 [ 0.2 ; 0.29 ] | <0.001 | 0.13 [ 0.08 ; 0.18 ] | <0.001 |
| RWT, mm | 0.00835 [ 0.00584 ; 0.01087 ] | <0.001 | 0.01099 [ 0.00858 ; 0.01341 ] | <0.001 | 0.00301 [ 0.00044 ; 0.00559 ] | 0.022 |
| LVPWD, mm | 0.21 [ 0.17 ; 0.26 ] | <0.001 | 0.17 [ 0.13 ; 0.21 ] | <0.001 | 0.17 [ 0.12 ; 0.21 ] | <0.001 |
| LVEDD, mm | 0.09 [ -0.07 ; 0.25 ] | 0.263 | -0.24 [ -0.4 ; -0.09 ] | 0.002 | 0.32 [ 0.16 ; 0.49 ] | <0.001 |
| LVEDV, ml | 2.02 [ 0.98 ; 3.07 ] | <0.001 | -1.5 [ -2.49 ; -0.5 ] | 0.003 | 4.15 [ 3.09 ; 5.2 ] | <0.001 |

**Supplementary Table 7. Multivariable linear regression analysis for the association of systolic and diastolic blood pressure with echocardiographic variables total cohort with additional adjustment for LV mass.** Adjustment was performed for age, sex, BMI, smoking, coronary artery disease, diabetes, atrial fibrillation, antihypertensive medication, and LV mass. Abbreviations as in *Table 2*.

|  | Systolic blood pressure | | Diastolic blood pressure | | Pulse Pressure | |
| --- | --- | --- | --- | --- | --- | --- |
|  | **ß per SD (95% CI)** | **p-value** | **ß per SD (95% CI)** | **p-value** | **ß per SD (95% CI)** | **p-value** |
| **FUNCTIONAL PARAMETERS** | | | | | | |
| LVEF, % | -0.06 [ -0.21 ; 0.09 ] | 0.465 | -0.24 [ -0.38 ; -0.1 ] | 0.001 | 0.12 [ -0.04 ; 0.28 ] | 0.128 |
| E/e' mean ratio | 0.26 [ 0.21 ; 0.32 ] | <0.001 | 0.08 [ 0.02 ; 0.14 ] | 0.005 | 0.32 [ 0.26 ; 0.38 ] | <0.001 |
| LV lateral e', cm/s | -0.22 [ -0.3 ; -0.14 ] | <0.001 | -0.46 [ -0.53 ; -0.38 ] | <0.001 | 0.07 [ -0.02 ; 0.15 ] | 0.126 |
| LV septal e’, cm/s | -0.2 [ -0.27 ; -0.13 ] | <0.001 | -0.32 [ -0.39 ; -0.25 ] | <0.001 | -0.02 [ -0.1 ; 0.05 ] | 0.524 |
| LASV, ml | 0.44 [ 0.18 ; 0.7 ] | 0.001 | -0.4 [ -0.64 ; -0.15 ] | 0.002 | 0.96 [ 0.68 ; 1.23 ] | <0.001 |
| **MORPHOLOGICAL PARAMETERS** | | | | | | |
| LVEDD, mm | -0.25 [ -0.36 ; -0.14 ] | <0.001 | -0.42 [ -0.53 ; -0.32 ] | <0.001 | -0.01 [ -0.12 ; 0.11 ] | 0.919 |
| LVEDV, ml | 1.01 [ 0.16 ; 1.86 ] | 0.02 | -2.17 [ -2.97 ; -1.37 ] | <0.001 | 3.24 [ 2.36 ; 4.12 ] | <0.001 |

**Supplemental Figure 1.** **Flow chart of participants from SHIP.** From a total of 6,753 subjects, 660 subjects were excluded due to clinical exclusion criteria and 1,485 subjects showed missing data. Consequently, 4,608 SHIP subjects were included for validation analysis.
